# Compositional Data Analysis of Relative Body Mass and Physical Function Using the Relative Shift Approach

**DOI:** 10.1002/oby.70307

**Published:** 2026-10-06

**Authors:** Daniel P. Beavers, Lauren S. Roe, Yutong Li, Katarina L. Dobrosky, Ian Janssen, Kristen M. Beavers

**Affiliations:** ^1^ Department of Statistical Sciences Wake Forest University Winston‐Salem North Carolina USA; ^2^ Department of Epidemiology University of Pittsburgh School of Public Health Pittsburgh Pennsylvania USA; ^3^ Department of Public Health Sciences and School of Kinesiology and Health Studies Queens University Kingston Ontario Canada; ^4^ Department of Internal Medicine, Section of Gerontology and Geriatric Medicine Wake Forest University School of Medicine Winston‐Salem North Carolina USA

**Keywords:** body composition, CoDA, compositional, isometric log‐ratio, relative shift

## Abstract

**Objective:**

The purpose of this study was to present the novel relative shift approach for compositional data analysis of body composition as a predictor for gait speed and to compare it to existing methods.

**Methods:**

Body composition was quantified from dual‐energy x‐ray absorptiometry in 145 older adults living with obesity. Gait speed was measured from a fast‐paced 400‐m walk. The associations between speed and body composition used separate least squares regression models with absolute (kg) mass and compositional methods based on isometric log‐ratio (ILR) transformations and the relative shift approach.

**Results:**

All methods found significant associations between gait speed and body composition. The ILR approach was fit three times, once for each component variable (lean, fat, and bone mass). Predictions differed for the same magnitude of 5% composition change due to total body mass differences (+4.4 [0.8 to 8.0] vs. +5.0 [1.2 to 8.8] cm/s). The absolute mass model ignored the relative nature of the components. The relative shift model required no transformation and provided consistent predictions for 5% change (+4.6 [1.0 to 8.2] cm/s).

**Conclusions:**

Our data suggest the relative shift model may provide simpler estimation and interpretation than ILR for models with compositional covariates.

**Trial Registration:**

ClinicalTrials.gov identifier NCT04076618

## Study Importance

- What is already known?
  ○ Compositional data analysis (CoDA) is a valid yet underutilized statistical method for analyzing data that quantify relative components of a whole, such as body composition.
  ○ CoDA methods can be difficult to implement and interpret in biomedical research studies, and novel, simpler approaches may provide greater accessibility and utility.
- What does this study add?
  ○ A new method for modeling compositional covariates called the relative shift approach is demonstrated to have several advantages over existing log‐transformation CoDA methods for quantifying the association between relative body composition and physical function.
  ○ Compared to existing CoDA and non‐CoDA models, a relative shift model requires no variable transformation, which makes model fitting and predictions easier to implement and interpret.
- How might these results change the direction of research or the focus of clinical practice?
  ○ Research that fails to account for compositional data when using relative measures may lead to inaccurate results or misinterpretation of associations. Widespread use of CoDA methods can help alleviate this, and the relative shift approach does not require specialized knowledge or mathematical tools to implement.

## 1 Introduction

Associations between body composition (e.g., fat, lean, and bone mass), morbidity, and mortality are well described in the literature. Often, components of body mass, such as kilograms of body fat and lean tissue, are modeled [1]; however, this approach ignores the compositional nature of the data. This is problematic when relative measures better explain differences in outcomes across individuals. For example, 15 kg of fat mass has a different health impact on a 150‐kg person compared to a 60‐kg person (10% vs. 25% of body mass, respectively). Data that define the relative parts of a whole are compositional data, that is, sets of relative quantities such as proportions that sum to a constant amount, usually 100% [2]. Compositional data frequently occur in obesity and energetics research, such as the relative composition of adipose, bone, and lean tissue in the human body [3] and the proportion of time that individuals engage in different intensities of movement [4, 5].

The study of compositional data presents challenges for analysis and interpretation because of the constraints on the values of variables: Proportions must be between 0 and 1, variables lack independence, and the sum of all variables is constant [2]. The unit sum constraint means that if a body is composed of k categories, then only k−1 of the categories may vary freely. Thus, it is imperative that compositional data be analyzed using tools that consider these complications [6].

Statistical approaches, broadly known as compositional data analysis (CoDA), have been developed to analyze compositional variables. A common CoDA approach, the isometric log‐ratio (ILR) transformation, constructs logarithmic transformations of ratios of proportions called pivot coordinates [7]. When used as covariates, these transformations permit estimation within the constraints of the unit sum but otherwise produce challenges in implementation and interpretation [8]. An ILR‐based regression model must be refit k times to identify important variables, and the interpretation of regression model findings requires complex methods where changes in outcomes are predicted based on transformed changes in ratios of the composition. These predictions are nonlinear; changes in proportions have different impacts on the outcome depending on the starting point of each component. A more useful approach would avoid complicated transformations of variables requiring specialized software, provide linear associations, and require a single fit of the model. In this paper, we introduce a previously defined and novel alternate CoDA approach called the relative shift (RS) method [9]. We argue the RS approach provides easier implementation and interpretation of CoDA models, making CoDA more intuitive and accessible.

## 2 Methods

### 2.1 Study Design and Sample

We compared approaches for CoDA using cross‐sectional baseline data from the INVEST in Bone Health Trial [10] from 145 men and women aged 60–85 living with overweight or obesity. Participants were recruited and randomized into one of three interventions to determine if weighted vest use or resistance exercise training could prevent bone loss associated with weight loss [11]. The study was approved by the institutional review board of Wake Forest University School of Medicine, and all participants provided written informed consent.

### 2.2 Study Measures

All measures included here were collected at baseline. Demographic information was self‐reported. Weight was collected via standardized scale. Body composition (including fat, lean, and bone mass) was quantified using dual‐energy x‐ray absorptiometry (DXA). Gait speed was obtained from a fast 400‐m walk test as an indicator of functional status [12].

### 2.3 Statistical Methods

The relationship between body composition and gait speed was modeled three ways as follows: (1) a non‐CoDA approach using body composition measured in its original scale of mass in kilograms; (2) a CoDA approach that transformed body composition proportions using ILR‐based pivot coordinates using the compositions R package [13]; and (3) the RS CoDA approach that used proportions of lean, fat, and bone as covariates [9]. The RS method fits the proportions as covariates in a linear regression model in their original unit scale using a zero‐intercept model. We describe the models in more detail in the online Supporting Information. All models used least squares regression with gait speed as the outcome and gender as a covariate, and regression assumptions were verified through the analysis of residuals. ILR models were fit three times, each time using a different body composition component as the numerator in the primary pivot coordinate. We used *F* tests to test the overall significance of body composition variables in each model using the multcomp R package [14].

## 3 Results

### 3.1 Sample Characteristics

Summary characteristics are in Table S1 [11]. The sample (*n* = 145) was predominantly female (*n* = 112, 74.7%), older (66.4 [SD = 4.6] years), and living with obesity (BMI 33.8 [SD = 3.2] kg/m [2]). In Figure 1 we present ternary plots of body composition by gender and gait speed. Men had higher proportions of lean mass (Figure 1a). Bone mass spanned from 1.8 to 4.2 kg (2.1%–3.8%), and fat mass ranged from 25 to 58 kg (29%–55%), while lean mass ranged from 34 to 73 kg (43%–67%). The mean (SD) gait speed of 131 (15) cm/s indicates participants were relatively functional; only 3.4% had gait speeds below 100 cm/s. Unadjusted gait speeds were lower for individuals with higher proportions of fat mass (Figure 1b).

**FIGURE 1:**
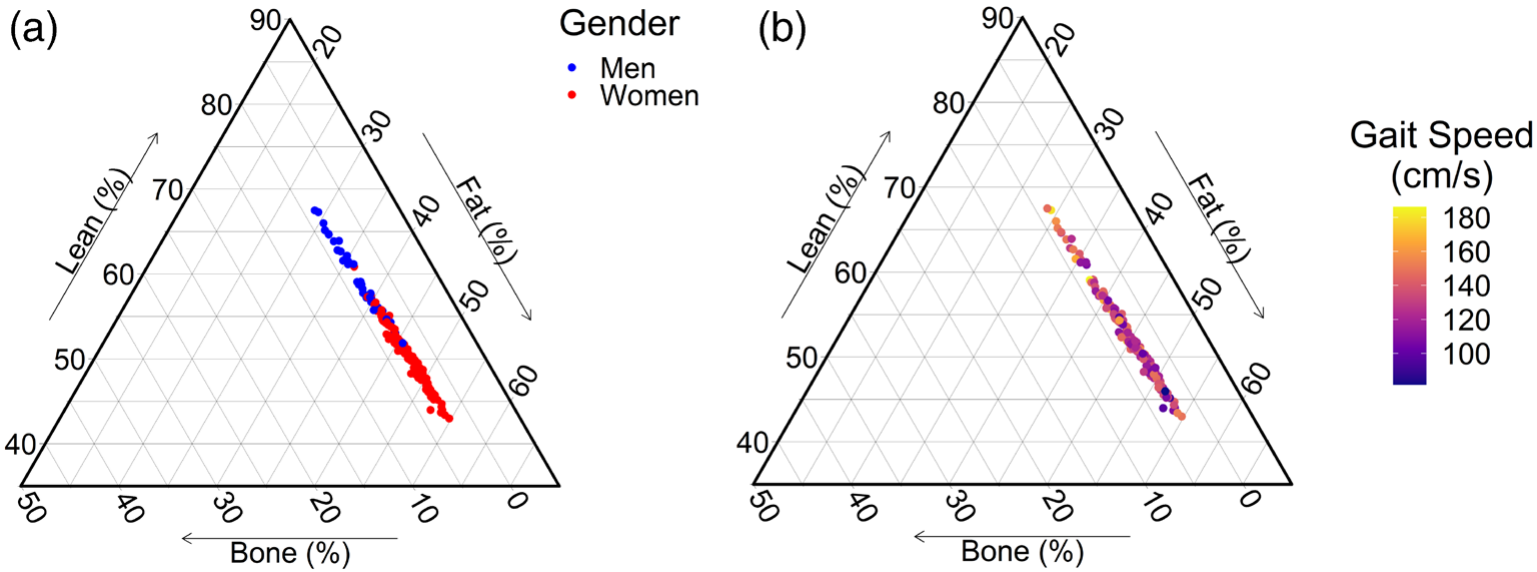
Ternary plots of body composition across gender (Figure 1a) and gait speed (Figure 1b). Each point lies at the intersection of the 3 body composition values for an individual. The topmost point in Figure 1a represents a man with 67.5% lean, 28.8% fat, and 3.7% bone mass.

### 3.2 Comparison of Models Associating Body Composition With Gait Speed

Table 1 displays the results of the three models. For the non‐CoDA approach, absolute fat mass was significantly associated with gait speed (−0.61 [95% CI: −1.04 to −0.18], *p* = 0.006), and the overall *F* test was marginally significant (*p* = 0.05). For the ILR‐pivot coordinates CoDA approach, there were no statistically significant relationships between function and primary body composition coordinates; however, *F* tests indicated an overall association between compositional variables and gait speed (*p* = 0.027). For the RS CoDA approach, the absolute values of the individual coefficients do not have interpretation, so we exclude statistical tests for individual terms. We found a significant association between compositional variables and gait speed (*p* = 0.029).

**TABLE 1:** Parameter estimates from modeling approaches.

Variable	Estimate (95% CI)	*p* for coefficients	*p* for compositional variables
Model 1: Absolute mass model			
Intercept	143.4 (106.9, 179.9)	< 0.001	
Lean mass	0.23 (−0.51, 0.97)	0.54	0.0497
Fat mass	−0.61 (−1.04, −0.18)	0.006	
Bone mass	1.21 (−8.70, 11.12)	0.81	
Gender	−2.47 (−15.84, 10.90)	0.72	
Model 2: Iterated ILR models			
Model 2a: Lean, fat, bone			
Intercept	145.7 (72.5, 218.9)	< 0.001	
PC: Lean versus fat + bone	−28.2 (−57.9, 1.5)	0.062	0.027
PC: Fat versus bone	7.9 (−22.8, 38.7)	0.61	
Gender	0.22 (−9.1, 9.5)	0.96	
Model 2b: Fat, lean, bone			
Intercept	145.7 (72.5, 218.9)	< 0.001	
PC: Fat versus lean + bone	28.2 (−57.9, 1.5)	0.062	0.027
PC: Lean versus bone	7.9 (−22.8, 38.7)	0.61	
Gender	0.22 (−9.1, 9.5)	0.96	
Model 2c: Bone, lean, fat			
Intercept	145.7 (72.5, 218.9)	< 0.001	
PC: Bone versus lean + fat	7.2 (−30.0, 44.5)	0.70	0.027
PC: Lean versus fat	−28.4 (−49.3, −7.5)	0.008	
Gender	0.22 (−9.1, 9.5)	0.96	
Model 3: Relative shift model			
Percent lean mass	161.2 (108.0, 214.4)	—	0.029
Percent fat mass	78.7 (26.3, 131.1)	—	
Percent bone mass	387.3 (−479.1, 1253.7)	—	
Gender	0.0 (−9.3, 9.3)	0.99	

Abbreviations: CI, confidence interval; ILR, Isometric log‐ratio; PC, pivot coordinate.

We used Table 1 results to predict gait speed changes for hypothetical body composition changes in order to demonstrate predictions in Figure 2. For the non‐CoDA absolute mass model, we estimated that a +4.8‐kg lean mass change, +0.2‐kg bone mass change, and −5.0‐kg fat mass change (holding overall body mass constant) would induce a gait speed 4.4 (0.0 to 8.8) cm/s (*p* = 0.05) faster. For the CoDA ILR‐pivot coordinate model, we used analogous changes of +4.8% lean, +0.2% bone, and −5% fat. This required identifying starting body compositions because cross‐sectional proportions, not their changes, must be ILR‐transformed for the model. Our first hypothetical person had 42.8%, 54.2%, and 3.0% lean, fat, and bone mass, respectively. The effect of the relative changes on gait speed was +4.4 (0.8 to 8.0) cm/s (*p* = 0.016). A second hypothetical person started with 58.8%, 37.6%, and 3.6% lean, fat, and bone mass, respectively, and the effect of the same relative changes on gait speed was +5.0 (1.2 to 8.8) cm/s (*p* = 0.011). Finally, we applied the +4.8% lean, +0.2% bone, and −5% fat change to the RS CoDA model, which predicted a mean gait speed change of +4.6 (1.0 to 8.2) cm/s (*p* = 0.013) regardless of starting body composition.

**FIGURE 2:**
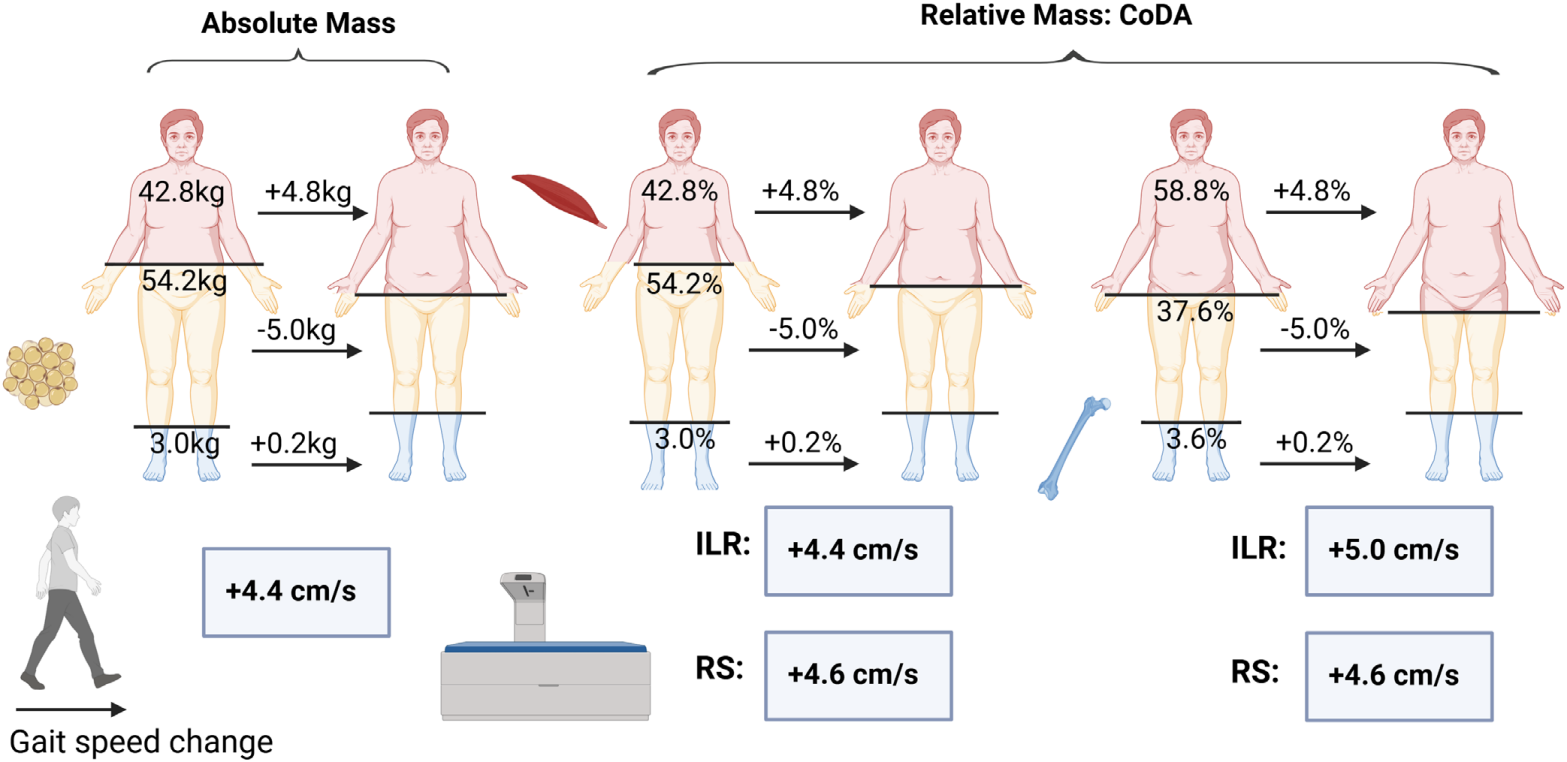
Estimated mean changes in gait speed based on changes in body composition: A comparison of 3 models. Absolute mass model estimates gait speed changes using lean, fat, and muscle mass in kg, adjusted for gender. ILR CoDA model uses pivot coordinates of relative body composition adjusted for gender. RS CoDA model uses percent body composition of lean, fat, and muscle mass as covariates in a zero‐intercept regression model, adjusted for gender. CoDA models are compared across 2 hypothetical individuals.

## 4 Discussion

This brief report demonstrated the RS approach to CoDA and its advantages relative to other statistical approaches. We found important differences in outcomes and ease of implementation across models. The ILR approach required specialized software to transform variables and repeated model fitting to quantify the effect of the composition variables on function; furthermore, using the model(s) for prediction required taking differences in transformed variables. The changes in gait speed for ILR depended on the starting relative body composition, so identical changes in relative measures yielded different outcome estimates. Both the absolute mass and RS models allow for untransformed variables in a single model (respectively). However, the absolute mass model ignored the relative nature of the data, and the increased uncertainty led to borderline statistically significant results. The RS approach allowed directly fitting body composition proportions using a zero‐intercept model without transformation, and the resulting predictions yielded narrower confidence intervals than the absolute model.

The ultimate utility is to provide descriptive and hopefully prescriptive quantities for improved health among individuals with overweight and obesity. The RS approach provides a novel and complementary approach to CoDA in ways that absolute volumes of composition and ILR‐based transformations cannot. If there is belief that body composition proportions may be associated with health outcomes [15], the RS approach demonstrates ease in both implementation and interpretation, enhancing its usefulness for researchers.

This paper compared the RS CoDA method with ILR CoDA and a non‐CoDA regression. However, important limitations exist. Users of the RS approach must use the k−1 degree of freedom statistical test for associations and must not use the tests for individual coefficients due to likely high multicollinearity. We used a three‐compartment model, but alternate imaging methods can quantify more specific tissue types [3]. Future work should identify general properties such as power between methods using statistical simulation. Also, researchers should consider models for longitudinal data analysis of changes in composition and outcomes. We view RS as a practical complement to, rather than a substitute for, ILR; RS may be preferred when prediction and communication of composition–outcome associations are the priority, whereas ILR remains preferable when complex geometric properties or specific log‐ratio hypotheses are required. Reporting both, as we do here, can serve as a useful strategy for assessing the robustness of compositional associations.

## Author Contributions

Concept and design, D.P.B., K.M.B., L.S.R.; acquisition of data, D.P.B., K.M.B.; analysis and interpretation of data, D.P.B., Y.L., K.L.D.; drafting of manuscript, D.P.B., K.M.B., K.L.D., Y.L.; critical revision, D.P.B., K.M.B., K.L.D., I.J., L.S.R., D.P.B. had full access to all the data in the study and takes responsibility for the integrity of the data and the accuracy of the data analysis. All authors approve the final version of the manuscript.

## Funding

This work was supported by the National Institute on Aging, Grants No. R01 AG059186 and K07 AG101495 (K.M.B.), K25 AG058804, T32 AG033534, F31 AG086010, and P30 AG021332. Jason Pharmaceuticals, a wholly owned subsidiary of Medifast Inc., made an in‐kind product donation for the meal replacements used in this study. Additionally, the authors gratefully acknowledge the use of the services and facilities of the Clinical Research Unit and Translational Imaging Program, funded by the National Center for Advancing Translational Sciences (NCATS), National Institutes of Health, through Grant Award Number UL1TR001420. The funders had no role in the design and conduct of the study; collection, management, analysis, and interpretation of the data; preparation, review, or approval of the manuscript; and decision to submit the manuscript for publication.

## Conflicts of Interest

The authors declare no conflicts of interest.

## Supporting information


**Table S1:** Demographic and clinical characteristics of study participants at baseline.

## Data Availability

The data that support the findings of this study are available from the corresponding author upon reasonable request.

## References

[1] K. M. Beavers , M. E. Miller , W. J. Rejeski , B. J. Nicklas , and S. B. Kritchevsky , “Fat Mass Loss Predicts Gain in Physical Function With Intentional Weight Loss in Older Adults,” Journals of Gerontology. Series A, Biological Sciences and Medical Sciences 68, no. 1 (2013): 80–86, 10.1093/gerona/gls092. PMC3598365 22503993

[2] J. Aitchison , “The Statistical Analysis of Compositional Data,” Journal of the Royal Statistical Society. Series B, Statistical Methodology 44, no. 2 (1982): 139–160, 10.1111/j.2517-6161.1982.tb01195.x.

[3] I. Janssen , E. Miller , E. Dzongowski , K. Miakisheva , and R. Ross , “A Compositional Analysis Study of Body Composition and Cardiometabolic Risk Factors,” Obesity 30, no. 8 (2022): 1699–1707, 10.1002/oby.23477. 35851760

[4] H. Sampasa‐Kanyinga , I. Colman , D. Dumuid , et al., “Longitudinal Association Between Movement Behaviours and Depressive Symptoms Among Adolescents Using Compositional Data Analysis,” PLoS One 16, no. 9 (2021): e0256867, 10.1371/journal.pone.0256867. PMC8409652 34469485

[5] D. Dumuid , T. E. Stanford , J. A. Martin‐Fernández , et al., “Compositional Data Analysis for Physical Activity, Sedentary Time and Sleep Research,” Statistical Methods in Medical Research 27, no. 12 (2018): 3726–3738, 10.1177/0962280217710835. 28555522

[6] C. Tilves , S. Peddada , and I. Miljkovic , “Body Composition Analyses Require Compositional Data Analytic (CoDA) Methods,” Obesity 29, no. 5 (2021): 783–785, 10.1002/oby.23132. PMC8340562 33759398

[7] J. J. Egozcue , V. Pawlowsky‐Glahn , G. Mateu‐Figueras , and C. Barceló‐Vidal , “Isometric Logratio Transformations for Compositional Data Analysis,” Mathematical Geology 35, no. 3 (2003): 279–300, 10.1023/A:1023818214614.

[8] D. Dumuid , J. Martín‐Fernández , S. Ellul , et al., “Analysing Body Composition as Compositional Data: An Exploration of the Relationship Between Body Composition, Body Mass and Bone Strength,” Statistical Methods in Medical Research 30, no. 1 (2021): 331–346, 10.1177/0962280220955221. 32940148

[9] G. Li , Y. Li , and K. Chen , “It's All Relative: Regression Analysis With Compositional Predictors,” Biometrics 79, no. 2 (2023): 1318–1329, 10.1111/biom.13703. PMC9767704 35616500

[10] R. M. Miller , D. P. Beavers , P. M. Cawthon , et al., “Incorporating Nutrition, Vests, Education, and Strength Training (INVEST) in Bone Health: Trial Design and Methods,” Contemporary Clinical Trials 104 (2021): 106326, 10.1016/j.cct.2021.106326. PMC8180512 33631359

[11] K. M. Beavers , S. D. Lynch , J. Fanning , et al., “Weighted Vest Use or Resistance Exercise to Offset Weight Loss–Associated Bone Loss in Older Adults: A Randomized Clinical Trial,” JAMA Network Open 8, no. 6 (2025): e2516772, 10.1001/jamanetworkopen.2025.16772. PMC12181796 40540267

[12] A. B. Newman , E. M. Simonsick , B. L. Naydeck , et al., “Association of Long‐Distance Corridor Walk Performance With Mortality, Cardiovascular Disease, Mobility Limitation, and Disability,” JAMA 295, no. 17 (2006): 2018–2026, 10.1001/jama.295.17.2018. 16670410

[13] K. G. van den Boogaart and R. Tolosana‐Delgado , “Compositions”: A Unified R Package to Analyze Compositional Data,” Computers & Geosciences 34, no. 4 (2008): 320–338, 10.1016/j.cageo.2006.11.017.

[14] T. Hothorn , F. Bretz , and P. Westfall , “Simultaneous Inference in General Parametric Models,” Biometrical Journal 50, no. 3 (2008): 346–363, 10.1002/bimj.200810425. 18481363

[15] C. M. Toomey , A. Cremona , K. Hughes , C. Norton , and P. Jakeman , “A Review of Body Composition Measurement in the Assessment of Health,” Topics in Clinical Nutrition 30, no. 1 (2015): 16–32, 10.1097/TIN.0000000000000017.

