# Supplementary material for: Compositional Data Analysis of Relative Body Mass and Physical Function Using the Relative Shift Approach
Source: Obesity (Silver Spring). 2026 Oct 6;34(11):2020–5. doi: 10.1002/oby.70307 (PMC13716194; doi:10.1002/oby.70307)
Supplement: Supplementary file 1 [file OBY-34-2020-s001.docx]

**SUPPORTING INFORMATION**

CoDA methods deal with approaches for handling data that are typically identified as a set of relative nonnegative variables whose sum is 1. For example, for an individual with total body mass $m$, we may represent an individual’s body adipose tissue mass, $w_{1}$; bone mass, $w_{2}$; and lean tissue mass $w_{3}$. Because of differences in height, sex, age, etc, it may be of interest to consider the relative rather than absolute mass of different body components. Often researchers construct proportions to quantify relative measures of components of a set: $x_{1}=w_{1}/m$, $x_{2}=w_{2}/m$, and $x_{3}=w_{3}/m$. We would describe the $x$’s as compositional because they sum to 1, and each $x_{i}$ represents the relative contribution of each tissue type to the whole rather than the individual tissue mass. A similar application is the relative proportion of the day children spend in various types of physical activity at school, especially when the duration of wear of accelerometers or length of school day differs across individuals. Unit-sum compositional variables are easily constructed in most software packages without requiring additional tools, but functions such as “acomp” from the “compositions” software package, among others, in R are available.

Analyses of compositional data use specialized methods because among the full set of $k$ compositional variables, only $k-1$ have uncertainty; mathematically, it is true that $x_{k}=1-\sum_{i=1}^{k-1} x_{i}$, which therefore could not vary if the other $k-1$ values were known. To overcome some of these challenges, compositional variables are often transformed for use in statistical models; one of the most widely used approaches in obesity research is the use of the isometric logratio (ILR) to construct what are known as pivot coordinates.^5,8^ The transformation deals with the unit sum constraint by taking the $k$ proportions and constructing $k-1$ orthonormal coordinates, which in a regression model can quantify the effect on an outcome of increasing/decreasing one component of the composition at the expense of decreasing/increasing other components. The general formula for constructing the $i$th coordinate from the $k$ compositional variables, $(x_{1},\ldots,x_{k})$, is

$$z_{i}=\sqrt{\frac{k-i}{k-i+1}}\ln\left( \frac{x_{i}}{\sqrt[k-i]{\prod_{j=i+1}^{k} x_{j}}} \right)$$

which is performed across all variables to build a vector of $k-1$ pivot coordinates. Fortunately software packages such as *compositions* in R can be used.^13^ The resulting $k-1$ variables can be fit as covariates in statistical models to weigh the contribution of one component on the outcome relative to all the others:

$$\begin{aligned} y=\phi_{0}+\sum_{j=1}^{p} \phi_{j}w_{j}+\sum_{i=1}^{k-1} \beta_{i}z_{i}+\varepsilon,\#\left( 1 \right) \end{aligned}$$

where $\phi_{0},\ldots,\phi_{p}$ are the intercept and coefficients for $w_{1},\ldots,w_{p}$ covariates, respectively, $\beta_{1},\ldots,\beta_{k-1}$ are the coefficients for the pivot coordinates, and $\varepsilon$ is the random variability term, typically assumed to be Gaussian. In such a model, the association of the compositional variable with the outcome is tested using the null hypothesis:

$$\begin{aligned} H_{0}:\beta_{1}=\ldots=\beta_{k-1}=0. \#\left( 2 \right) \end{aligned}$$

This can help answer questions such as whether the proportion of time spent in moderate to vigorous activity (MVPA) is more important for childhood obesity relative to the time spent in sleep, sedentary, or light activity.

The interpretation of the coefficient for an ILR pivot coordinate variables is typically limited to the first coordinate because it alone contains all the compositional variables. Thus, only the first coordinate that uses the ratio of the first variable versus the remaining $k-1$ has utility. Subsequent coordinates can only quantify the contribution of a variable compared to the remaining variables, omitting the numerator coordinates of earlier coordinate variables; this requires refitting the model up to $k$ times, each time modifying the numerator of the first pivot coordinate, which can be burdensome when $k$ is large.

An alternative proposal for determining the relationship between compositional predictors and an outcome is the *relative shift* (RS) approach.^9^ Using the RS model, the proportion variables $x_{1},\ldots,x_{k}$ are fit without transformation as covariates in a linear regression model:

$$\begin{aligned} y=\sum_{j=1}^{p} \phi_{j}w_{j}+\sum_{i=1}^{k} \beta_{i}x_{i}+\varepsilon,\#\left( 3 \right) \end{aligned}$$

where $\phi_{1},\ldots,\phi_{p}$ and $w_{1},\ldots,w_{p}$ are defined as in (1), $\beta_{1},\ldots,\beta_{k}$ are the coefficients for compositional variables $x_{1},\ldots,x_{k}$, and $\varepsilon$ is the random error. In the RS model, the association of the compositional variable with the outcome is tested using the null hypothesis: $\begin{aligned} H_{0}:\beta_{1}=\ldots=\beta_{k}.\#\left( 4 \right) \end{aligned}$

Note that this hypothesis does not require equivalence to zero. Regression assumptions are checked using standard methods such as residual plots for both models (1) and (3).

To overcome complications from the unit sum constraint that challenges most CoDA methods, model (3) is fit without an intercept term. This creates a model in which parameter estimates represent the effect of each respective component on the outcome, but the scale of measurement of the coefficients depends on the measurement scale of the outcome. Consequently, the coefficients are not compared to a null hypothesized 0 for statistical testing but rather to each other. This allows for the simultaneous use of all $k$ compositional variables at a time during statistical inference in a single model. Also, it can easily be implemented in linear regression functions in statistical software packages, unlike ILR methods, which require variable transformations, extra computational tools, and iterated refitting of the model.

An alternative but identical RS approach is to fit only $k-1$ covariates from the model in (3), where each $x_{i}$ is a proportion: $\begin{aligned} y=\phi_{0}+\sum_{j=1}^{p} \phi_{j}w_{j}+\sum_{i=1}^{k-1} \beta_{i}x_{i}+\varepsilon,\#\left( 5 \right) \end{aligned}$

In model (5), the association of the compositional variable with the outcome is tested using the null hypothesis: $\begin{aligned} H_{0}:\beta_{1}=\ldots=\beta_{k-1}=0.\#\left( 6 \right) \end{aligned}$

This approach includes an intercept and fits only $k-1$ compositional variables, and all inference and predictions in (3) and (5) are identical; also, null hypotheses (4) and (6) are identical. For the sake of space, we only use model (3) for this manuscript.

**Table S1:** Demographic and Clinical Characteristics of Study Participants at Baseline.

| **Characteristic** | **Overall,**  **N = 145***^1^* | **Male,**  **N=38 (26%)** | **Female,**  **N=107 (74%)** |
| --- | --- | --- | --- |
| Age (years) | 66.3 ± 4.5 (60,80) | 68.1 ± 4.2 (61,77) | 65.6 ± 4.4 (60,80) |
| Body Mass Index (kg/m^2^) | 33.8 ± 3.2 (27.3,40.0) | 34.1 ± 3.2 (28.4,39.3) | 33.6 ± 3.3 (27.3,40.0) |
| DXA-Acquired Total Body Composition |  |  |  |
| Total Body Mass (kg) | 92 ± 13 (68, 122) | 104 ± 11 (86,122) | 88 ± 10 (68,117) |
| Fat Mass (kg) | 41 ± 7 (25, 58) | 39 ± 8 (26,53) | 41 ± 7 (25,58) |
| Lean Mass (kg) | 48 ± 9 (34, 73) | 62 ± 6 (49,73) | 44 ± 5 (34,56) |
| Bone Mass (kg) | 2.7 ± 0.6 (1.8, 4.2) | 3.4 ± .4 (2.6,4.2) | 2.4 ± .3 (1.8,3.5) |
| Relative Body Composition |  |  |  |
| Fat Mass (%) | 44 ± 6 (29, 55) | 37 ± 5 (29,47) | 47 ± 4 (36,55) |
| Lean Mass (%) | 53 ± 6 (43, 67) | 59 ± 4 (50, 67) | 50 ± 4 (43, 61) |
| Bone Mass (%) | 2.9 ± 0.4 (2.1, 3.8) | 3.3 ± .4 (2.6, 3.8) | 2.8 ± .4 (2.1, 3.7) |
| Gait Speed (cm/s) | 131 ± 17 (82,186) | 138 ± 19 (98,186) | 129 ± 16 (82,172) |
| *^1^*n (%); Mean ± SD (Min,Max) | |  |  |
